# Eating Frequency in European Children from 1 to 96 Months of Age: Results of the Childhood Obesity Project Study

**DOI:** 10.3390/nu15040984

**Published:** 2023-02-16

**Authors:** Vanessa Jaeger, Berthold Koletzko, Veronica Luque, Dariusz Gruszfeld, Elvira Verduci, Annick Xhonneux, Veit Grote

**Affiliations:** 1 Division of Metabolism and Nutritional Medicine, Department of Pediatrics, Dr. von Hauner Children’s Hospital, LMU University Hospitals, 80337 Munich, Germany; 2 Paediatrics Research Unit, Universitat Rovira I Virgili-IISPV, 43201 Reus, Spain; 3 Serra Hunter Fellow, Universitat Rovira I Virgili-IISPV, 43201 Reus, Spain; 4 Neonatal Department and Neonatal Intensive Care Unit, Children’s Memorial Health Institute, 04-730 Warsaw, Poland; 5 Department of Paediatrics, Vittore Buzzi Children’s Hospital, University of Milan, 20154 Milan, Italy; 6 Groupe Santé CHC, Bd. Patience et Beaujonc 2—(B), 4000 Liège, Belgium

**Keywords:** eating frequency, feeding frequency, formula feeding, infants, children, eating behavior, overweight, meal, snack, time-of-day

## Abstract

We aimed to investigate the eating frequency (EF) in children over age, and examined the influence of country, sex, feeding mode and weight status on EF. We used the dietary data of the Childhood Obesity Project, which comprised European children from five countries. Dietary data of 3-days weighed and estimated records were available monthly from 1 to 9 and at 12-, 24-, 36-, 48-, 60-, 72- and 96-months old. Generalized additive mixed effects models were used to estimate EF trajectories with EF as outcome and applying age splines. Additionally, the models were further adjusted for country, feeding mode, sex or weight status. Data from 1244 children were analysed. EF was highest at 1 month with on average 7.3 ± 1.9 feeds per day, and fell to 5.1 ± 1.1 eating occasions at the age 96 months. Night feeding was similarly often than day feeding at 1 month but declined thereafter. Significant differences in EF were observed between countries (*p* < 0.05), with the highest EF in Poland, and between infant feeding modes, with a higher EF in breastfed than non-breastfed infants (*p* < 0.05). Sex and body weight were not associated with EF. Despite the importance of EF towards total energy intake, no association with weight status was found.

## 1. Introduction

Eating behavior typically includes the amount as well as the type of foods and beverages consumed. However, eating frequency (EF) as the third component of eating behavior is less frequently examined [1]. Recently, more studies have examined EF because it seems to affect appetitive, digestive and metabolic processes [2], and thus may influence health and wellbeing.

The eating pattern of adults typically consists of three main meals and one to two snacks per day [3]. In children and infants, such a structured eating behavior is less well established. EF is expected to change throughout childhood, along with marked changes of development and dietary needs: infants receive either breastmilk or formula, along with complementary feeding introduced usually between the 4th and 6th month [4]. At around 12-months-old, children are able to eat similar type of foods to the other family members [5]. With increasing age, infants and children acquire added motor, cognitive, social and language skills which may influence EF. For instance, motor skills enable the child to eat without further major help from the 2nd and 3rd year of life onwards. With increasing age, children are more interested in peers and want to be similar to them, which can affect eating behavior [6].

Few studies have examined the EF in infants and children. The available studies focus on specific eating occasions (EO) such as snacking [7,8,9]. However, to study regular habits, EFs of the whole day are needed. In addition, many studies in infants and children comprise of short observation periods or are of cross-sectional design [10,11,12]. Since the eating behavior of young children is likely to track into older ages [13] it is important to examine the longitudinal effects of the EF during the whole day, ideally prospectively from infancy to childhood. 

It is not known whether factors such as sex or cultural differences influence EF. Data on associations of EF with weight status in children are inconsistent. A meta-analysis of cross-sectional studies in children and adolescents concluded that further longitudinal studies are needed [14]. It has been suggested that infant feeding mode (breastfed vs. non-breastfed) may influence the eating behavior in infants [15], but data are limited.

In this prospective longitudinal study, we studied the EF of European children during the age from 1-to-96-months-old. We aimed to examine the EF trajectory evolution with age, during the time-of-day, and potential associations with infant feeding mode (breastfeeding vs. non-breastfeeding), sex, country of residence and weight status. 

## 2. Methods

### 2.1. Study Design and Population

This analysis used dietary data collected as part of the Childhood obesity Project (CHOP) initiated as a double-blinded randomized controlled trial to test the effects of early protein intake on growth and later obesity risk. Some 1678 healthy, singleton infants born at full-term were enrolled at a median age of 2 weeks and no later than the age of 8-weeks-old [16,17]. Infants received isoenergetic study formulas with different protein contents, and isoenergetic follow-on formulas with different protein contents were offered after introduction of complementary feeding until the age of 12 months. In addition to the intervention, an observational group of infants fully breastfed for at least 3 months was included. Infants were recruited from Belgium (Brussels, Liege), Germany (Munich, Nuremberg), Italy (Milano), Poland (Warsaw) and Spain (Tarragona, Reus). Written informed consent was received from all legal guardians prior to enrolment, and additional assent from children at the follow-up at 96 months of age. All time points with dietary records were included for this analysis, namely, monthly nutritional data for the first 9 months of the infant’s life, and at 12-, 18-, 24-, 36-, 48-, 60-, 72- and 96-months-old. The trial was conducted in accordance with the Declaration of Helsinki, approved by the ethical committee from each study site and registered at clinical trials (NCT00338689).

### 2.2. Dietary Assessment

Dietary data for the aforementioned time points were collected using food records for three consecutive days, including one weekend day. The dietary records were completed as proxy reports by parents or legal caretakers. Food records from 1 to 24 months of age were weighed using food scales (Unica 66,006 food scale; Soehnle, Murrhardt, Germany). From 36 months onwards, foods and leftovers were weighed wherever possible. Otherwise, an atlas of food pictures was used to support estimation of portion sizes as described previously [18]. Breastfeeding was entered as an EO with zero calories because quantitative intake data were collected only in a subgroup of Italian children (weighing infants before and after breastfeeding).

Data were entered in a software specifically dedicated for this study, where nutritional data were based on the German food database BLS 2.3 (Bundeslebensmittelschlüssel). If needed, the database was enriched by information from product labels, manufacturers or national food databases of participating countries. Nutritional values of foods were updated for analysis to BLS 3.1. Quality checks were performed for each food record by trained nutritionists following standard operating procedures [19,20].

Misreporting of food intakes was assessed for all time points with weight measurements by comparing individual cut-off values with the ratio of reported energy intakes to estimated energy requirements as described in detail elsewhere [21]. Individual reports were identified as misreporting if the individual ratio was above or below the cut-off value, but were not excluded from the analysis as recommended [22].

### 2.3. Eating Frequency (EF)

EF was assessed as the total number of EOs (foods and beverages) consumed within a day. Water intake was not considered as an EO because water consumption was typically reported as a summarized intake during the day. Further beverage intakes with an energy density (amount of energy (kcal) per gram of food (g)) less than 0.01 (analogous to [23]), which typically include unsweetened tea, were not considered to avoid potential bias; i.e., some children prefer water as a beverage, and others prefer tea. Food records, in which EF was not assigned to a particular point in time (e.g., stating “during day”) were excluded.

EOs were collected either as exact time-of-day (<36 months of age) or were assigned according to pre-defined categories (≥36 months of age). These categories include breakfast, lunch, supper and morning, afternoon and evening snacks as described previously [24]. Additionally, all EOs were seen as one EO when time was less than 30 min for lunch and supper, or less than 15 min for breakfast and snacks. This time differentiation was used to include foods such as dessert as one meal, which are typically eaten at lunch or supper.

### 2.4. Covariates

Height or length, respectively, as well as weight during the first 2 months of age and at 3, 6, 12, 24, 36, 48, 60, 72 and 96 months were assessed according to the procedures of the World Health Organization (WHO) Growth reference study [25]. Children were categorized as being normal or overweight according to the International Obesity Task force (IOTF) cut-off values, available for children aged 24 months and older [26]. Parental education was collected by questionnaire at baseline and classified according to the International Standard Classification of Education [27] into low, medium and high levels of education. Smoking in pregnancy, maternal age at child’s birth, birth weight, week of birth, country and sex were collected by questionnaire. Start of complementary feeding was defined through questionnaires and dietary records.

### 2.5. Statistical Analysis

All subjects with dietary data from ≥one observation time point were included in the analysis. The data were divided for a main analysis and a sub-analysis. The main analysis included all dietary records except those from breastfed infants, where total energy and nutrient intakes could not be calculated. Data of breastfed infants were added to the sub analysis. EF, total energy (kcal/day) and nutrient (g/day) intakes were averaged over the three-day dietary records. Descriptive data are presented as arithmetic mean (standard deviation) for continuous variables and as counts (%) for categorical variables.

EF trajectories by age were estimated by generalized additive mixed-effects models. Mixed effects model enable the estimation of repeated measurements and allow for missing values at observation time points, whereas the generalized additive mixed model is particular suitable to model non-linear effects via splines such as age over time. Furthermore, trajectory modelling by regression analysis in contrast to crude averages allow to estimate confidence intervals by taking into account the EF correlation of the same child. A regression analysis was estimated with EF as outcome and age in months (penalized cubic spline; “cs”) as exposure. Additionally, the model contained a random intercept for each study participant, a random slope over age and an autocorrelation term for age in months (“CorCAR1”). The inclusion of an autocorrelation term was necessary to consider residual serial correlation from repeated measurements of the same child. Additional models were estimated to examine country, feeding mode, sex or weight status differences in EF. These models additionally included country, feeding type (breastfeeding vs. non-breastfeeding), sex or weight status (normal weight vs. overweight) as a covariate, and an additional interaction term was added. This term contained a spline with age in months interacting with either country, feeding mode, sex or weight status. The interaction allowed different shapes between countries, sexes, feeding modes or weight groups, and additionally examined differences over time. Country or sex differences in EF were estimated for all ages, whereas IOTF cutoffs for weight status were available only from 24 months onwards [26]. Differences between feeding mode were estimated during the 1st year of life. Results of the regression analyses were plotted using predicted average values of EF over time and EF over time, country and feeding type, respectively. Models were graphically checked by inspection of residuals.

For the analysis of EF by time-of-day, EF was either examined according to EOs or by time-of-day (in hours) in percentage of total records. The observation time points were further summarized to plot daytime differences between countries and feeding modes. 

Several sensitivity analyses were performed. Firstly, a sensitivity analysis was calculated by the exclusion of EOs contributing less than 5% towards total energy intake (TEI), and secondly, a sensitivity analysis was performed by exclusion of study participants in which TEI deviated more than three standard deviations from the average TEI for each time point. Thirdly, a sensitivity analysis was performed to examine the effect of continuous BMI z-score instead of cut-off values. Cut-off values facilitate the visual presentation; on the other hand, they do face some drawbacks such as loss of information and thus loss of statistical power [28]. Lastly, we examined the effect of the intervention on EF by including the intervention type as covariate variable into the model. All analyses were performed in R (R Foundation for Statistical Computing, version 4.2.2., Vienna, Austria) [29] in addition to the packages “mgcv” and “ggplot2”. Results were considered as significant at *p* < 0.05. 

## 3. Results

The main analysis of this study included 1244 healthy children from 1 to 96 months of age. About a quarter of the children were in the observational breastfed group. The sub analysis also included all children with breastfeeding, which resulted in 1370 children (Supplementary Online Material—Table S1). In addition to the number of breastfed children, the main and sub-analysis had similar study characteristics, and the characteristics of the main study population are reported in the following. Thirty-seven percent of the children each belonged to the intervention and control group. Children participated on average in 10.9 out of 17 dietary follow-up visits, with 13% of children participating in all of the 17 follow-up visits. Most children were recruited in Spain (27%), followed by Italy (25%), Germany and Poland (17% each), and Belgium (13%). Around half of the parents of participating children had a medium education level, and the average age of the mother at birth of the study child was 30 years (Supplementary Online Material—Table S2).

Dietary intakes by age is shown in Table 1. Average EF decreased from 7.3 to 5.1 from 1 to 96 months of age, whereas TEI and dietary energy density increased. TEI by weight declined from 1 to 96 months of age at time points with weight measurements. Average nutrient (g) intakes increased with age, but the distributions of energy from nutrients changed by age, with an increase of energy from carbohydrates from 44%E to 53%E from 1 to 7 months, to 49%E at 96 months. Similarly, energy from protein increased from 9%E to 17%E in children from 1 to 18 months to 15%E at 96 months. Instead, energy from fats decreased from 44%E to 36%E from 1 to 96 months. Possible misreporting of TEI was highest at 96 months (underreporting: 25%) and 12 months (overreporting: 33%). Children classified as underreporting of TEI had on average a lower EF than children classified as overreporting or children with plausible report of TEI. In contrast, children classified as overreporting had on average a higher EF than children with plausible report or underreport. Around 15% of children aged 2 to 8 years were overweight at a given time point with highest frequency at 8 years (23% of the children) and lowest frequency at 2 years of age (6% of the children).

**Figure 1:**
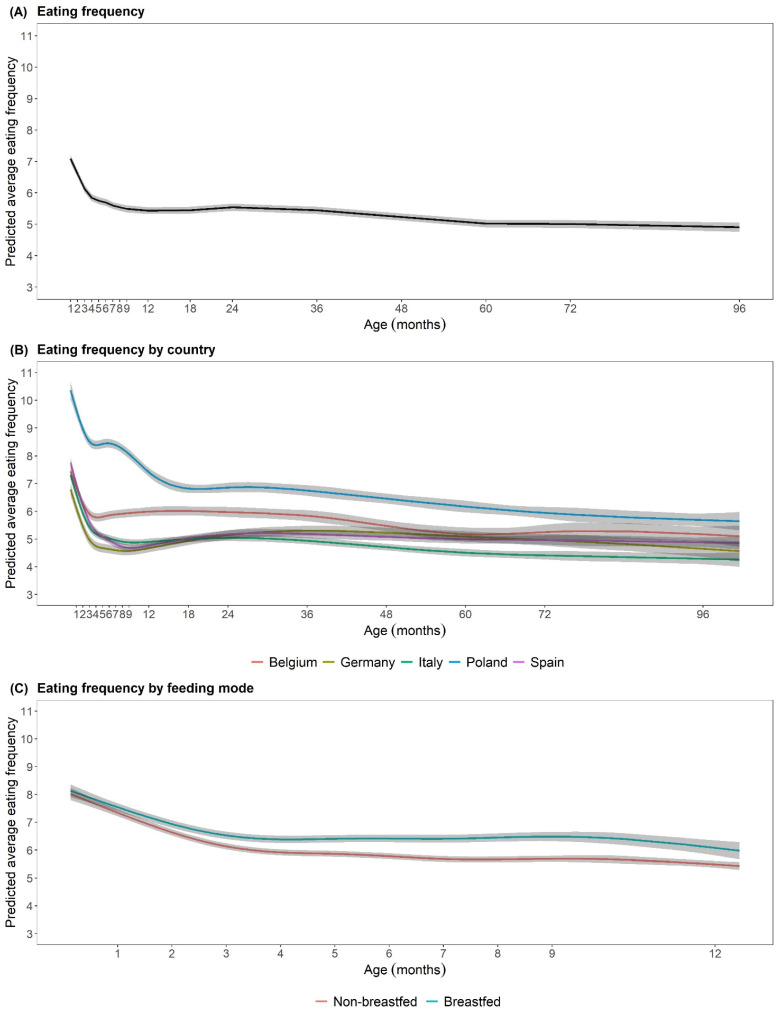
(**A**) Predicted average eating frequency by age (*n* = 1244). (**B**) Predicted average eating frequency by age and country (n_Belgium_ = 163, n_Germany_ = 214; n_Italy_ = 314; n_Poland_ = 214; n_Spain_ = 339). (**C**) Predicted average eating frequency by feeding mode (*n* = 1370, whereas 536 infants were breastfed at a given time point). All models are estimated by generalized additive mixed-effects models with EF as outcome and a spline for age in months, a spline for age in months and country and a spline for age in months and feeding mode, respectively, as exposure.

The average predicted EF by age is shown in Figure 1. The difference of the predicted EF records (Figure 1A) compared to the raw average EF (Table 1) were very similar. From the 1st to 3rd months a steep decline in EF was observed; complementary feeding was on average introduced at the end of the 4th month (18.9 weeks). Thereafter, the decrease in EF was decelerated or constant, whereas EF started to increase slightly with 24 to 36 months and declined thereafter. The calculated EFs by country are shown in Fig 1B. Polish children showed the highest average EF due to a regular consumption of tea in the first months. For all countries, the highest EF was seen at 1 months, with a steep decline thereafter, and the lowest EF at 96 months of age. The most pronounced differences in EF between countries were seen during the first months of life, while EF were more similar at later ages. Differences between breastfed and non-breastfed infants during the 1st year of life are shown in Figure 1C. In total, 536 infants were breastfed at a given time point (Supplementary Online Material—Table S1). Infants were grouped as breastfed as long as breastfeeding persisted and otherwise grouped as non-breastfed along with formula-fed infants from the main analysis. Infants showed a higher EF for the duration of breastfeeding than non-breastfed infants, particularly pronounced from around 6 months onwards. From that time point onwards, breastfed infants were fed on average two times more often than non-breastfed infants. Sex and body weight status were not associated with EF (*p* > 0.05).

**Figure 2:**
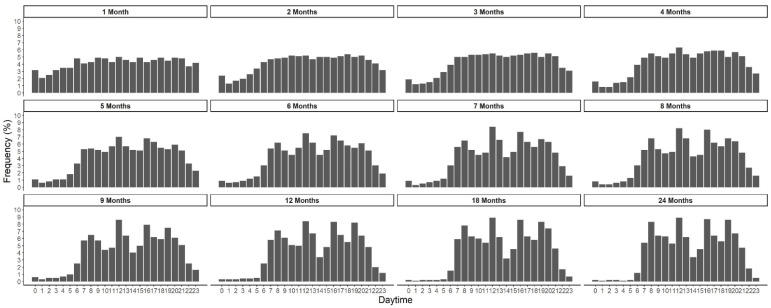
Eating frequency by time-of-day in percentage from total records and by age in months (*n* = 1244).

**Figure 3:**
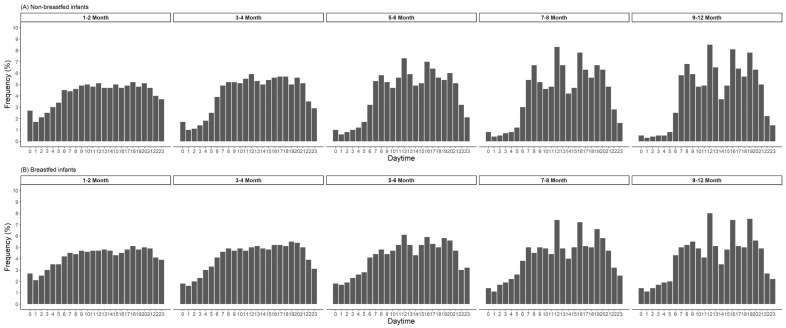
Eating frequency by time-of-day in percentage from total records and by age groups and feeding mode (*n* = 1370 infants, whereas 536 infants were breastfed at given time point).

**Figure 4:**
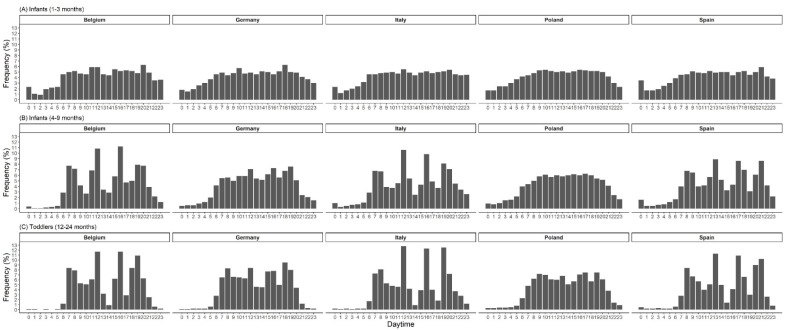
Eating frequency by time-of-day in percentage from total records and by age groups (**A**–**C**) and country (n_Belgium_ = 163, n_Germany_ = 214; n_Italy_ = 314; n_Poland_ = 214; n_Spain_ = 339).

EF by time of the day in children from 1 to 24 months is shown in Figure 2. At 1 month of age, the feeding distribution is almost equally distributed over 24 h. From 2 months onwards, the frequency of feedings during nighttime decreased steadily, and from around 6 months onwards peaks of feeding/eating appear at typical times during morning, midday and evening (Figure 2). EF by daytime showed earlier peaks of preferred meal times in non-breastfed compared to breastfed infants. Nighttime feeding was prevalent longer in breastfed infants (Figure 3). From around 4 months onwards, breastfed infants were fed twice as often during the night (10 pm to 5 am) than non-breastfed infants. With the introduction of complementary feeding, country differences in daytime EF were observed (Figure 4). The distribution is equally distributed during daytime, especially in Poland, though also in Germany. However, the eating distribution between 12–24 months in Germany is more pronounced during the afternoon and evening. Instead, in Belgium, Italy and Spain, there are noticeable peaks of EF. In Belgium and Italy, the first peak is visible between 7 to 8 am followed by noon, 4 pm and 7 pm. In Spain, all peaks were shifted 1 or 2 h later. Children aged 3 to 8 years showed a regular eating pattern with three meals (90% of the children; 8% of the children consumed more than three meals) and a regular snacking pattern with one, two or more than two snacks (28%, 47% and 24% of the children, respectively). Almost all children consumed at least one snack during the afternoons (95%), 62% of the children consumed at least one snack during the mornings and some during the evenings (18%). Children in Poland and Germany consumed (two or more than two times) a snack during the mornings (Poland: 43% and Germany: 20%) and the afternoons (Poland: 66% and Germany: 42%) more frequently. 

Several sensitivity analyses were carried out. One sensitivity analysis was performed by exclusion of dietary records deviating at least three standard deviations from the average TEI. The EF by age was similar to the main analysis. The second sensitivity analysis was performed by exclusion of EOs contributing only 5% or less towards TEI. The EF was slightly lower compared to the main analysis but was more similar towards 96 months of age (Supplementary Online Material—Figure S1). As a third sensitivity analysis, we examined BMI as a continuous variable, but found similar results to BMI as cut-off values. Lastly, we examined the effect of the original intervention on EF, but found a statistically significant higher EF over time only in the observational breastfed arm (*p* < 0.05). Particularly, the EF was elevated in the observational arm during the first year of life (Supplementary Online Material—Figure S2).

## 4. Discussion

This study examined the dietary data of 1244 healthy children from five European countries and from 1 to 96 months of age. We assessed the average EF by age and found the highest EF at 1 month followed by a steep decline until complementary feeding was introduced, and a stable-to-slightly decreasing EF until 96 months with the lowest EF. EF trajectories were shaped by feeding mode, with a higher EF in breastfed than non-breastfed infants, and by country, with the highest EF in Poland. Depiction of EF by daytime revealed a change in EF during the first 9 months of life from a rather uniform distribution at 1 months to peaks of preferred meal times from 4 months onwards. Daytime differences in EF were visible between countries as soon as complementary feeding was introduced. Breastfed infants were more often and longer fed during the night than non-breastfed infants. No differences in EF between children with different weight status or between sexes were found.

The WHO recommends to breastfeed on demand during the first about 6 months of life, which means to breastfeed whenever the infants signals the desire to drink—day or night [30]. Feeding on demand is also applicable for formula-fed infants [31]. In our study population, we found a slightly higher frequency of day compared to night feeding in the first month. The night-feeding frequency dropped from month to month, particularly in non-breastfed infants. A study in breastfed infants found night feeding to be higher before midnight and after 4 am [32], similarly to our study. We found the highest average feeding frequency to be at 1 month. Thereafter, the feeding frequency declined rapidly until 3 months, with a rather constant feeding frequency until 6 months. A similar feeding trajectory was found in breastfed Australian infants [33]. In this study, the authors additionally examined the breastfeeding duration and total 24 h milk intake, and found that the duration of feeding decreased steadily but the total milk intake remained constant. They concluded that breastfeeding becomes more efficient between the 1st and 3rd month of lactation, which may be related to an increasing stomach capacity of the infant. The feeding frequency in Polish infants was noticeable higher than in infants from remaining countries due to regular tea consumption from the 1st month onwards. Some institutions such as the WHO [34] recommend the addition of extra fluids, preferably water, only to non-breastfed children, as formula feeding compared to breastfeeding might not provide enough liquids. The consumption of tea or sugary drinks, however, should be limited due to its low nutrient density [35].

With introduction of complementary feeding, the WHO recommends to practice responsive feeding [5,35]. The principle of responsive feeding is based on reciprocity between the caregiver and the child. In particular, it means to feed children depending on the hunger and satiety cues of the child. The caregiver responds to these cues in an emotionally supportive and age-appropriate way, and the child experiences a predictable response to these signals [36]. Responsive feeding supports the child to self-regulate food intake and reduces overeating [37]. The WHO reported theoretical calculations as a benchmark for an appropriate number of feedings in children aged 6 to 23 months. These are four to five meals daily and one–two snacks if desired for formula-fed children, depending on the energy density and usual amounts consumed [35]. A meal may consist of milk feeds, other foods or a combination of both. In breastfed infants, the number of feeds from breastfeeding needs to be considered and were subtracted by the WHO from the recommended number of meals. Thus, the WHO [5] reported a benchmark of two–three meals at 6–8 months and of three–four meals at 9–11 months, depending on energy density and usual amounts consumed. Additionally, one–two snacks may be consumed if desired. Comparison of our results with the calculations by the WHO are difficult since WHO separated between meals and snacks. Nonetheless, the average energy density and energy requirements on which the required meals are calculated are comparable to the WHO.

In our study population, complementary feeding was on average introduced at the end of the 4th month. Changes in eating behavior were visible in the 24 h-food distribution as peaks of preferred meal timing started to appear. A cross-sectional study in American infants and children examined EF based on a meal and snack pattern instead of times, and found a meal and snack pattern emerging in the age group of 7–8-month-old infants and was established in 9–11-month-olds [38]. When looking in our analysis at the 24-h food distribution stratified by country, we found large country differences starting as early as from the 4th month onwards. The country differences we observed might suggest that the cultural component seems to be more important than purely physiological aspects to determine time and frequency of eating, as previously proposed by Chiva [3].

We found a higher EF in breastfed compared to non-breastfed infants. Complementary feeding was introduced later in breastfed (mean 5th month) compared to formula-fed infants (mean 4th month). However, this does not entirely explain the difference in EF, as the difference in EF was stable until the end of the 1st year of life. The higher EF in breastfed infants was particularly visible during nighttime, and is likely to result in different sleeping patterns between breastfed and non-breastfed infants during the entire lactation period. 

Most studies examining EF in children focused on children aged 2 years or older. The average daily EF in Portuguese children aged 3–9 years was 5.7, with on average three meals and three snacks [10]; in New Zealand children aged 2 years, the average EF was 5.5, with most children eating four–seven times per day [39], and in British children aged 4–10 years, the daily EF was 4.9 times per day [11]. The EF of these studies are comparable to our results; however, comparison is hampered due to different definitions of an EO and due to summarized age ranges as EOs decrease with age. Furthermore, EF from above studies were examined in countries not examined in our analysis.

We observed a decrease in average EF throughout the examined age range, as well as a decrease in variation with the smallest variation at 8 years (SD = 1.1). A possible explanation for the observed decrease in EF might be the improved gastric capacity and the decreased requirements for energy and nutrients per kilogram body weight [40,41]. Furthermore, the EF between children becomes more and more similar. A possible reason might be that with increasing age, the day becomes more structured and eating more institutionalized, with fixed times for meals and snacks in day care or school. Additionally, in older children, peers are becoming more important, and the eating behavior of peers might influence the eating behavior of the child to a greater extend [6].

A further major influencing factor of EF might be by the rate of gastrointestinal motility as gastric motility is linked to the sensations of hunger, appetite and satiety [42,43]. Gastric emptying, defined as the rate at which gastric contents are delivered to the duodenum, is regulated, for instance, by the volume or energy density of the ingested foods [43]. Thus, large EOs or EOs with a high energy density may slow down sensations of hunger and affecting EF. In addition, it has been found that gastric emptying is faster in breastfed than formula-fed infants [44,45]. A possible explanation might be the different nutrient composition. For instance, the milk fat content in breastmilk seems to increase throughout a feeding, whereas in infant formula the composition is uniform throughout a feeding [44,46].

We examined how EF is affected by weight status and sex differences, and found neither differences in EF between children with overweight and normal weight nor between boys and girls. A meta-analysis [14] of cross-sectional studies examining EF and overweight in children and adolescences found a beneficial effect of EF (highest vs. lowest EF category) on body weight, which was only significant in boys. However, the results of the meta-analysis are hampered by a high heterogeneity between study results and of the cross-sectional design of the studies included. In contrast, we performed a longitudinal study, which may reduce the risk of chance findings. We used EF as outcome and weight status as exposure variable, which is in reverse to previous studies and further limits comparison. Our results suggest that EF is of minor relevance in the overweight and obesity development. Future studies examining the overweight and obesity risk should focus on other dietary aspects than on the EF in children. 

## 5. Strength and Limitations

Our study benefited from the long prospective observation period from 1 to 96 months of age, which provided a detailed EF trajectory, particularly during the 1st year of life. Furthermore, the longitudinal study design allows not only the capacity to examine between-subject variations, but also within-subject variations, which strengthens evidence. We used 3-days weighed and estimated dietary records, which allowed a more accurate description of dietary data than other methods. Our study population comprised children from five European countries. This increased, on the one hand, the generalizability of our results, and additionally enabled the capacity to study country and cultural differences, respectively. On the other hand, it limits study comparison by age due to the different definitions used in younger and older children. A further limitation is that beverages could not be examined on their own. Beverages may influence metabolic and digestive processes differently than solid foods, and may have a different influence on eating behavior and health outcomes [1]. However, beverages are difficult to collect as beverages are often consumed over longer time periods, and are thus difficult to assign to one particular point in time. Furthermore, it may be easier to forget to report beverages. Due to these reasons, we did not perform a trajectory analysis of beverages only. Future studies need to focus on beverages to explicitly collect beverage consumption. Lastly, the frequency of breastfeeding, but not the volume of breastfeeding, was reported. Due to this, we could not validate the intake in breastfed infants, and we thus analysed breastfeeding only in a sub-analysis. The volume of breastfeeding is usually received by weighing the infant before and after the feeding. This method was not possible to apply in a large study such as ours, and was performed only in a subgroup of Italian children.

## 6. Conclusions

This study examined EF in European infants and children from 1 to 96 months of age and found a varying EF as children grow as well as between countries, feeding mode and during daytime. These findings, particularly during the first months of life, are relevant for health care professionals in supporting parents and their infants. Although EF is an important contributor towards TEI, it did not affect overweight risk in children. It seems that the cultural differences found in the EF and its distribution throughout the day may be more important than purely physiological factors.

## Figures and Tables

**Table 1:** Dietary intake by age in participants from 1 to 96 months of age (*n* = 1244).

Age in Months	*n*	Eating Frequency	Energy		Carbohydrates		Protein		Fat		Misreporting **		Energy Density
			Kcal/day	Kcal/kg/day *	%E	g/day	%E	g/day	%E	g/day	Under- Report *	Over- Report *	
1	616	7.3 ± 1.9	526 ± 117	154 ± 42	44 ± 2	57 ± 12	9 ± 2	12 ± 4	47 ± 3	27 ± 6	---	---	0.7 ± 0.3
2	833	7.1 ± 2.2	571 ± 125	---	44 ± 2	62 ± 13	9 ± 2	13 ± 4	47 ± 3	29 ± 6	---	---	0.7 ± 0.3
3	839	6.6 ± 2.1	600 ± 136	99 ± 22	44 ± 2	65 ± 15	9 ± 2	14 ± 4	47 ± 3	31 ± 6	104 (12)	86 (10)	0.7 ± 0.3
4	808	6.4 ± 2.1	640 ± 136	---	46 ± 4	72 ± 17	9 ± 2	15 ± 5	45 ± 5	31 ± 6	---	---	0.7 ± 0.3
5	815	6.4 ± 2.2	685 ± 147	---	49 ± 6	83 ± 21	10 ± 3	18 ± 6	40 ± 6	30 ± 7	---	---	0.7 ± 0.3
6	812	6.3 ± 2.3	731 ± 176	93 ± 21	52 ± 6	94 ± 24	11 ± 3	20 ± 7	36 ± 6	29 ± 8	39 (5)	205 (25)	0.7 ± 0.3
7	784	6.1 ± 2.1	772 ± 172	---	53 ± 6	101 ± 24	13 ± 3	24 ± 8	34 ± 6	29 ± 8	---	---	0.8 ± 0.4
8	778	6.1 ± 2.1	812 ± 183	---	53 ± 6	106 ± 24	13 ± 3	26 ± 8	33 ± 6	30 ± 8	---	---	0.8 ± 0.4
9	783	6.2 ± 2.2	838 ± 200	---	53 ± 6	109 ± 26	13 ± 3	28 ± 9	33 ± 6	30 ± 9	---	---	0.8 ± 0.5
12	829	5.9 ± 1.9	896 ± 208	91 ± 22	52 ± 7	115 ± 28	15 ± 3	33 ± 10	33 ± 6	32 ± 9	47 (6)	273 (33)	1.0 ± 0.7
18	742	5.8 ± 1.7	1042 ± 246	---	50 ± 7	128 ± 32	17 ± 3	42 ± 12	33 ± 6	38 ± 10	---	---	1.2 ± 0.9
24	778	5.9 ± 1.7	1120 ± 283	90 ± 24	49 ± 7	136 ± 33	16 ± 3	45 ± 13	34 ± 6	42 ± 12	61 (8)	171 (22)	1.2 ± 0.9
36	556	5.8 ± 1.6	1234 ± 303	83 ± 22	50 ± 7	150 ± 37	16 ± 3	47 ± 12	34 ± 6	46 ± 13	36 (6)	95 (17)	1.3 ± 1.0
48	528	5.5 ± 1.4	1329 ± 304	78 ± 20	50 ± 7	162 ± 35	15 ± 3	50 ± 13	35 ± 6	51 ± 14	29 (5)	73 (14)	1.4 ± 1.1
60	483	5.3 ± 1.4	1404 ± 325	72 ± 19	50 ± 7	172 ± 40	15 ± 3	52 ± 13	35 ± 6	54 ± 14	48 (10)	57 (12)	1.4 ± 1.0
72	520	5.2 ± 1.2	1481 ± 325	68 ± 17	50 ± 6	183 ± 39	15 ± 3	55 ± 13	35 ± 6	57 ± 14	60 (12)	43 (8)	1.4 ± 1.0
96	444	5.1 ± 1.1	1596 ± 364	57 ± 16	49 ± 7	191 ± 42	15 ± 3	60 ± 15	36 ± 6	63 ± 18	109 (25)	14 (3)	1.5 ± 1.1

Values are presented as mean ± standard deviation or as counts (percentage). * Weight in kg was not collected at months 2, 4, 5, 7, 8, 9 and 18; ** Misreporting was calculated by the ratio of reported energy intakes and estimated energy requirements [21]. Abbreviation: %E = Nutrient intake in percentage of energy intake.

## Data Availability

Data available on request due to ethical restrictions.

## Supplementary Materials

The following supporting information can be downloaded at: https://www.mdpi.com/article/10.3390/nu15040984/s1, Table S1: Participant flow of enrolled infants with dietary data at baseline and follow-ups; Table S2: Characteristics of participating children and their parents; Figure S1: Average predicted eating frequency by sensitivity analyses and age. All models are estimated by generalized additive mixed effects models with EF as outcome and a spline for age in months; Figure S2: Average predicted eating frequency by intervention (n_control_ = 463; n_experimental_ = 469; n_observational_ = 438). Statistical significant differences over age only in the observational arm (breastfed infants). No statistical significant difference between experimental and control group. Model is estimated by generalized additive mixed effects models with EF as outcome and a spline for age in months and age in months and intervention group.
