# Supplementary material for: Eating Frequency in European Children from 1 to 96 Months of Age: Results of the Childhood Obesity Project Study
Source: Nutrients. 2023 Feb 16;15(4):984. doi: 10.3390/nu15040984 (PMC9958886; doi:10.3390/nu15040984)
Supplement: Supplementary file 1 [file nutrients-15-00984-s001.zip › Supplementary material - Table S1.pdf]

Table S1 Participant flow of enrolled infants with dietary data at baseline and follow-ups.

Number of subjects with dietary data at baseline and follow-ups																	
**Age (months)**	**1***	**2***	**3**	**4**	**5**	**6**	**7**	**8**	**9**	**12**	**18**	**24**	**36**	**48**	**60**	**72**	**96**
	1,049	1,213	1,188	1,144	1,131	1,098	1,018	990	971	946	797	820	576	535	492	527	447
**Number of subjects excluded due to…**																	
**Inaccurate eating frequency** or illness**	6	1	9	12	18	18	20	25	36	30	28	29	19	7	9	7	3
**Breastfeeding*****	427	379	340	324	297	269	215	188	152	87	27	13	1	--	--	--	--
**Study population N = 1,244**																	
**Subjects**	616	833	839	808	815	812	784	778	783	829	742	778	556	528	483	520	444

*Infants were enrolled during the first 2 months of life. **Inaccurate eating frequency defined as missing time information or as food records, in which EF was not assigned to one particular point in time, e.g. “during day”. *** Number of breastfed infants were included in the sub analysis resulting in a total of 1,370 infants and a total of 536 breastfed infants at a given time point.
