# Supplementary material for: Eating Frequency in European Children from 1 to 96 Months of Age: Results of the Childhood Obesity Project Study
Source: Nutrients. 2023 Feb 16;15(4):984. doi: 10.3390/nu15040984 (PMC9958886; doi:10.3390/nu15040984)
Supplement: Supplementary file 1 [file nutrients-15-00984-s001.zip › Supplementary material - Table S2.pdf]

Table S2 Characteristics of participating children and their parents

**Characteristics**	**Main study population** (N = 1,244)	**Sub study population*** (N = 1,370)
Girls, n (%)	624 (50)	689 (50)
Country, n (%)		
Belgium	163 (13)	182 (13)
Germany	214 (17)	235 (17)
Italy	314 (25)	370 (27)
Poland	214 (17)	222 (16)
Spain	339 (27)	361 (26)
Intervention group, n (%)		
Experimental group (formula with low protein)	458 (37)	469 (34)
Control group (formula with high protein)	461 (37)	463 (34)
Observational group (Breastfed Infants)	325 (26)	438 (32)
Highest education level reached by one of the parents**, n (%)		
High	382 (31)	438 (32)
Middle	646 (52)	705 (51)
Low	214 (17)	225 (16)
Birthweight of the child, m ± SD	3,290 ± 350	3,296 ± 345
Week child was born, m ± SD	40 ± 1	40 ± 1
Mother´s age at child´s birth in years, m ± SD	30 ± 5	30 ± 5
Smoking in pregnancy, n (%)	431 (35)	462 (34)
Week child was introduced to complementary foods, m ± SD	18.9 ± 5.1	19.2 ± 5.0

Missing information: Smoking in pregnancy, n = 3; Education parents, n= 2; Age mother, n = 1; Week child born, n = 2; Introduction complementary feeding, n= 32. * Sub study population include breastfed infants. ** Education level classified according to International Standard Classification of Education [26].
