# Supplementary figures and images for: Eating Frequency in European Children from 1 to 96 Months of Age: Results of the Childhood Obesity Project Study
Source: Nutrients. 2023 Feb 16;15(4):984. doi: 10.3390/nu15040984 (PMC9958886; doi:10.3390/nu15040984)

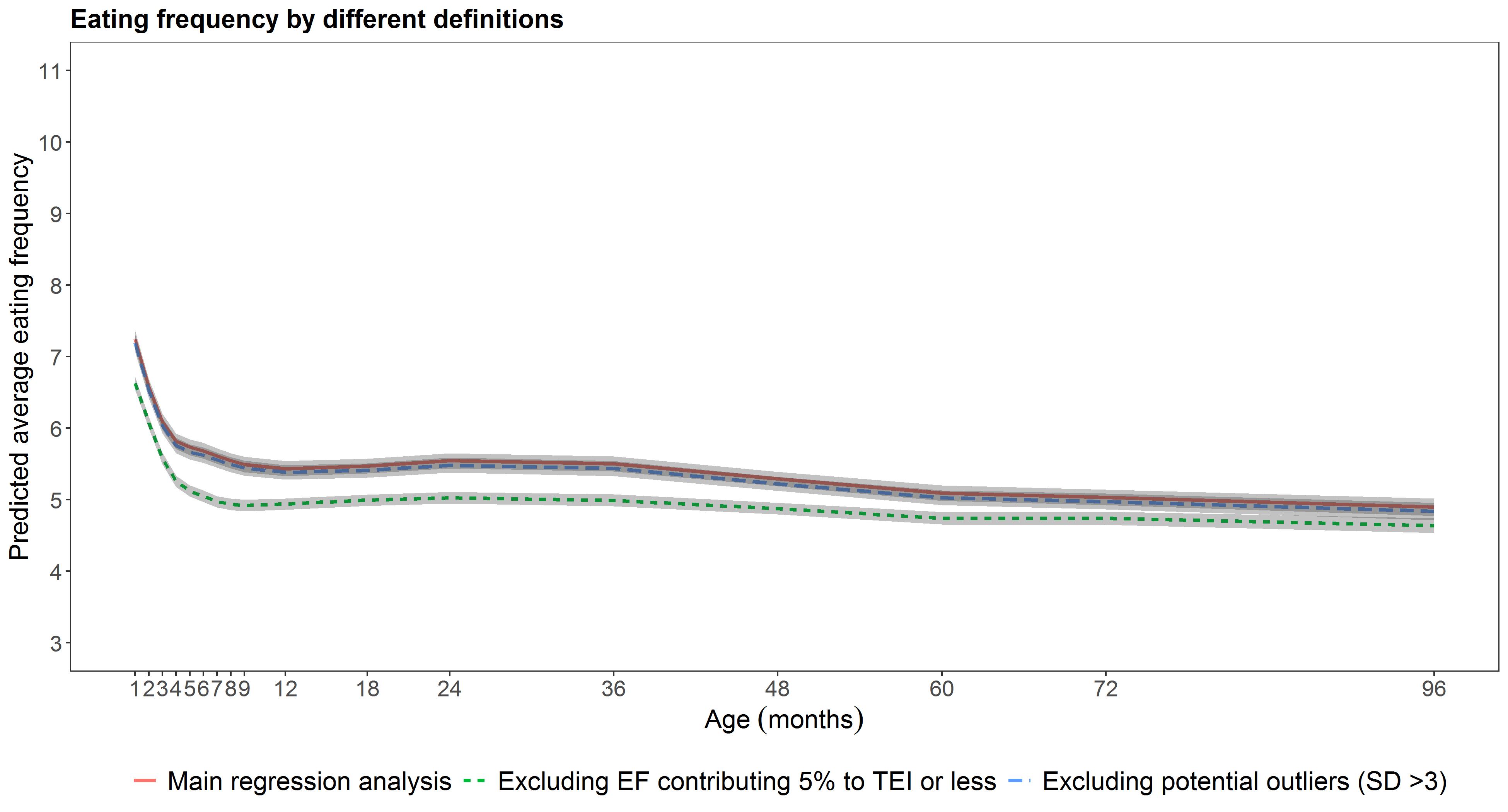

Supplement: Supplementary file 1 [file nutrients-15-00984-s001.zip › Supplementary material - Figure S1.jpeg]

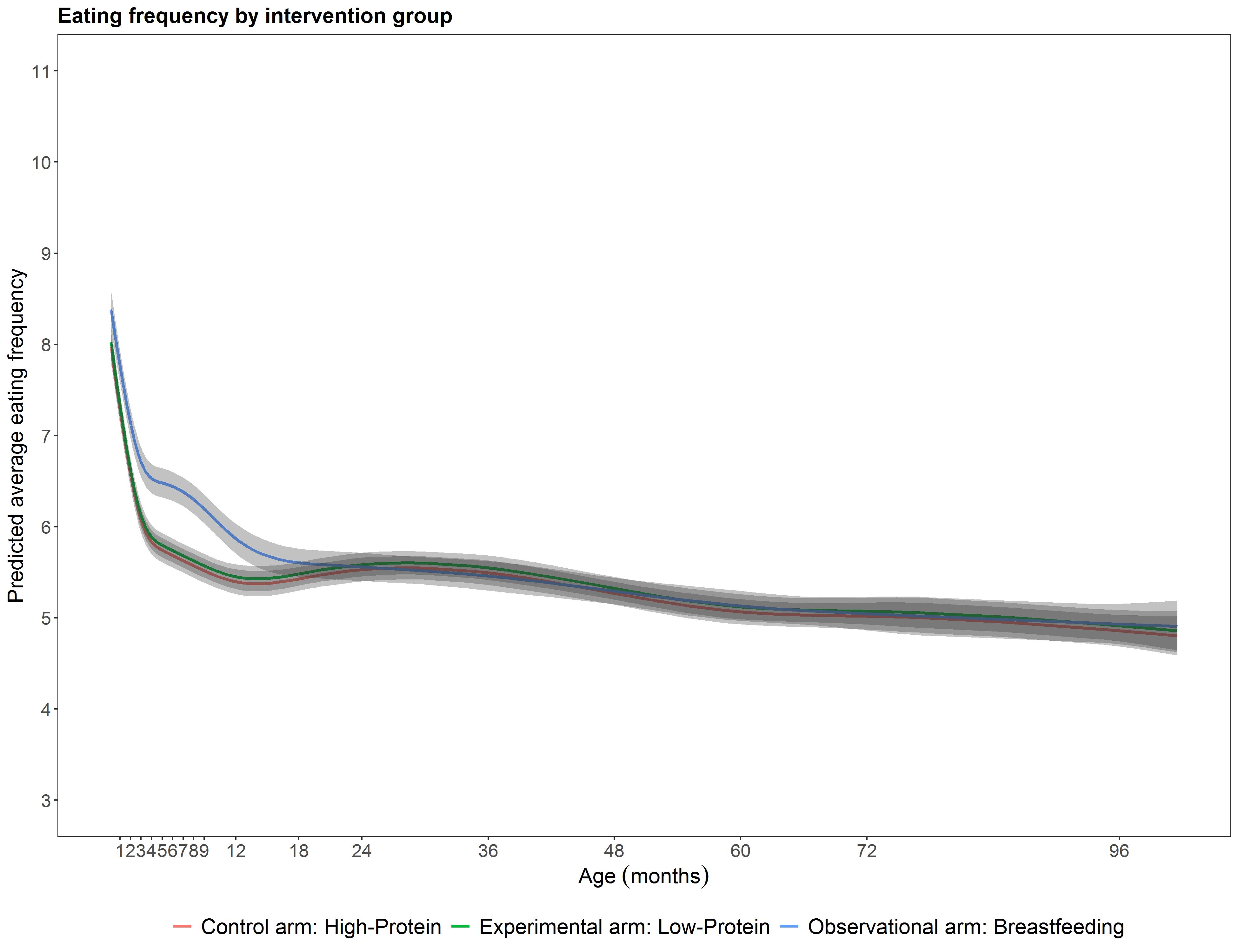

Supplement: Supplementary file 1 [file nutrients-15-00984-s001.zip › Supplementary material - Figure S2.jpeg]
